# Nonreciprocal Charge Transport in an Iron‐Based Superconductor with Broken Inversion Symmetry Engineered by a Hydrogen‐Concentration Gradient

**DOI:** 10.1002/advs.202524270

**Published:** 2026-02-15

**Authors:** Takayuki Nagai, Yukito Nishio, Jumpei Matsumoto, Kota Hanzawa, Hidenori Hiramatsu, Hideo Hosono, Tsuyoshi Kimura

**Affiliations:** ^1^ Quantum‐Phase Electronics Center (QPEC) and Department of Applied Physics University of Tokyo Tokyo Japan; ^2^ Materials and Structures Laboratory, Institute of Integrated Research Institute of Science Tokyo Yokohama Japan; ^3^ MDX Research Center For Element Strategy, Institute of Integrated Research Institute of Science Tokyo Yokohama Japan; ^4^ Research Center for Materials Nanoarchitectonics National Institute for Materials Science Tsukuba Japan

**Keywords:** concentration gradient, iron‐based superconductor, nonreciprocal charge transport, space inversion symmetry, vortex motion

## Abstract

The breaking of spatial inversion symmetry in condensed matter gives rise to intriguing physical properties, such as ferroelectricity, piezoelectricity, spin‐momentum locking, and nonreciprocal responses. Here, we propose that a concentration gradient, which often persists as a quasi‐stable nonequilibrium state with long relaxation times in solids, can serve as a general platform for inversion symmetry breaking. We demonstrate this concept in an epitaxial thin film of the hydrogen‐doped SmFeAsO (Sm1111:H) superconductor with a depthwise hydrogen‐concentration gradient introduced via an optimized topotactic reaction. This film exhibits nonreciprocal charge transport—that is, current‐direction‐dependent resistance—which serves as a key signature of broken inversion symmetry. A pronounced nonreciprocal signal emerges in the vicinity of the superconducting transition, which we attribute to vortex‐motion nonreciprocity arising from an asymmetric pinning landscape created by the hydrogen‐concentration gradient. Owing to the high critical temperature of Sm1111:H, vortex‐origin nonreciprocity is observed above 40 K, representing the highest temperature reported to date among single bulk materials without an artificially hetero‐layered structure. Our findings establish concentration‐gradient engineering as a versatile and broadly applicable route for realizing inversion‐broken states in otherwise centrosymmetric hosts, opening pathways toward a broader landscape of odd‐parity‐driven functionalities.

## Graphical abstract

Concentration gradients can serve as a universal platform for breaking spatial inversion symmetry. We demonstrate this concept by observing nonreciprocal electrical transport—a sensitive probe of inversion‐symmetry breaking—in the iron‐based superconductor Sm1111 with an engineered hydrogen gradient. In this system, asymmetric vortex dynamics induce nonreciprocal charge transport, which persists to remarkably high temperatures owing to the high superconducting transition temperature.

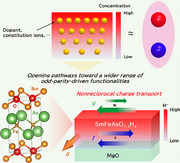

## 1 Introduction

Inversion symmetry (P) breaking is a fundamental ingredient in condensed matter, enabling practical functionalities and giving rise to exotic quantum phenomena. Representative examples include ferroelectricity and piezoelectricity, which emerge from broken P and underpin the operating principles of nonvolatile memories and sensors, driving widespread technological development [1, 2]. Concomitantly, P‐breaking activates antisymmetric spin‐orbit coupling, which in turn permits spin‐split band structures without magnetism, parity‐mixed non‐trivial superconductivity, and nonreciprocal transport and optical properties [3, 4, 5, 6, 7, 8]. Realizing a P‐broken platform is therefore a central objective in materials science.

Traditionally, inversion asymmetry has been achieved through several routes, including intrinsically noncentrosymmetric crystal structures, surfaces/interfaces/heterostructures [9, 10, 11, 12], externally applied electric fields [13], and strain gradients [14, 15]. Each route faces practical constraints. Noncentrosymmetric compounds are comparatively scarcer than centrosymmetric ones, and P‐related domains often cancel their intrinsic responses unless a poling field is applied or electrodes are fabricated within single domains. Interface‐based approaches are limited by material combinations and are sensitive to interfacial morphology. Field‐induced polarity frequently demands complex device structures (e.g., electric double‐layer transistors), while strain‐gradient‐induced displacements are typically small. These limitations motivate a more universal and controllable platform for P‐breaking.

Herein, we propose a symmetry‐based strategy in which a concentration gradient of dopant ions plays a crucial role in breaking P. In condensed matter, a concentration gradient frequently arises as a quasi‐stable state with long relaxation times. By symmetry, a concentration gradient transforms as a polar vector, identically to an electric polarization, and therefore breaks P. Once such a gradient is established, phenomena characteristic of P‐breaking are expected to emerge. To demonstrate this concept, we engineered a depthwise concentration gradient of doped hydrogen in the iron‐based superconductor SmFeAsO_1−_ *_x_* H*_x_* ​ (Sm1111:H) and examined nonreciprocal charge transport, a benchmark electrical signature that necessitates broken P. Engineering a concentration gradient thus converts a centrosymmetric host into a P‐broken platform. More broadly, this approach provides a general and chemically versatile paradigm for creating and controlling P‐breaking in condensed‐matter systems.

## 2 Conceptional Framework

### 2.1 Engineering Inversion‐Symmetry Breaking via Concentration Gradients

**FIGURE 1:**
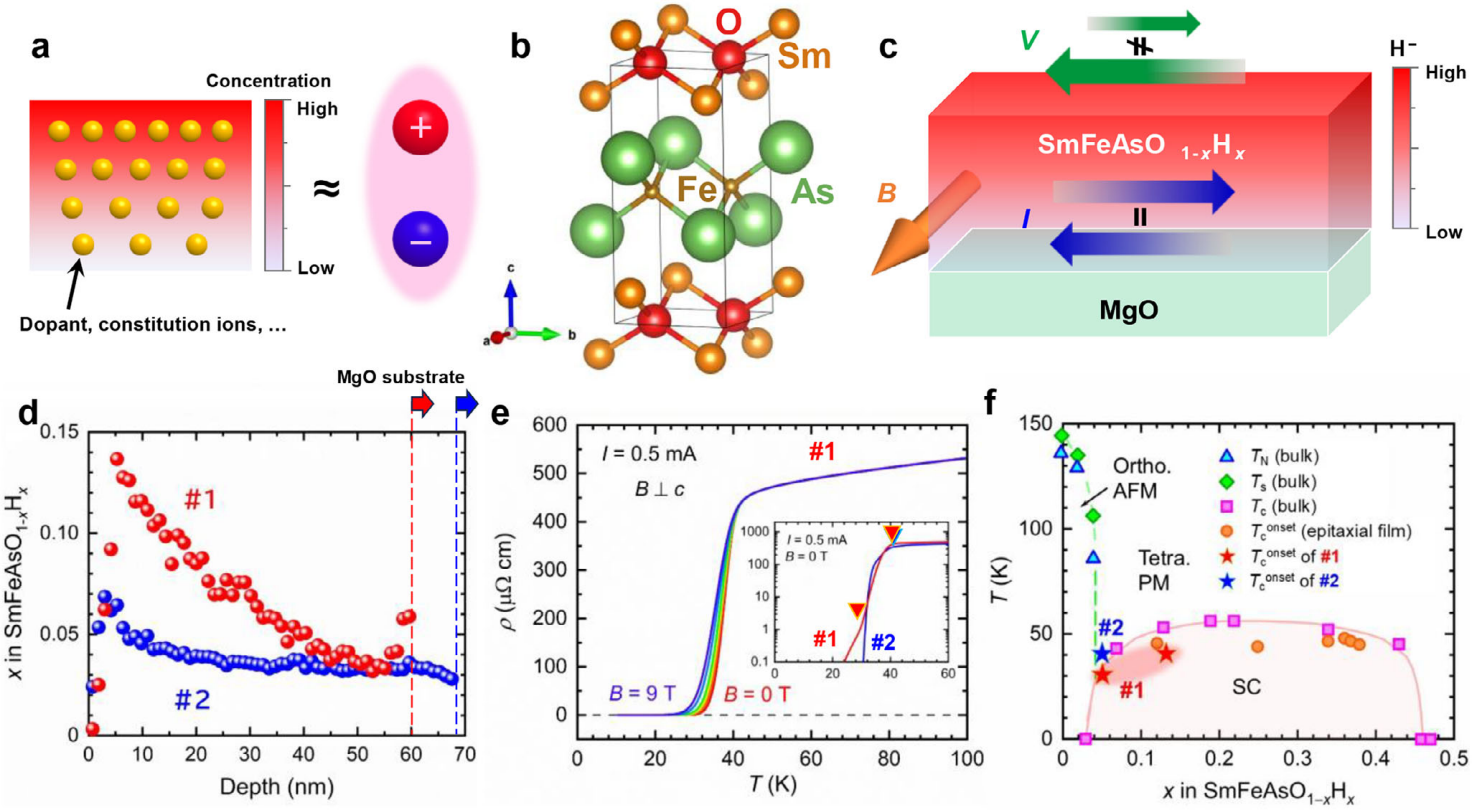
Concentration‐gradient‐driven inversion‐symmetry breaking and nonreciprocal charge transport, and basic properties of the prepared films. (a) Concept of inversion symmetry breaking driven by the concentration gradient **∇** *n*, which is symmetry‐equivalent to an electric polarization ***P***. (b) Schematic of the crystal structure of Sm1111; orange, red, brown, and green spheres indicate Sm, O, Fe, and As, respectively. The crystal structure was drawn using the software VESTA [64]. (c) Schematic illustration of nonreciprocal charge transport in the iron‐based superconductor Sm1111 with a hydrogen concentration gradient **∇** *n*_H_. *V*, ***B***, and ***I*** denote a voltage drop, a magnetic field, and an electric current, respectively. (d) Depth profile of the hydrogen content *x* in SmFeAsO_1−_ *_x_* H*_x_* measured by Second Ion Mass Spectrometry (SIMS). Dashed lines indicate the Sm1111/MgO interface. (e) Temperature dependence of the electrical resistivity *ρ* of sample #1 under in‐plane magnetic field *B* = 0, 1, 2, 3, 6, and 9 T. The applied current is 0.5 mA. The inset shows the logarithmic plot of *ρ*–*T* curves for samples #1 and #2 under zero magnetic field. Filled triangles denote $T_{c}^{\mathrm{onset}}$, defined by the onset of the decrease in *ρ*. In sample #1, the triangle at lower temperatures represents an anomaly with a shoulder‐like structure in the transient regime. (f) Phase diagram of SmFeAsO_1−_ *_x_* H*_x_* as a function of *x*. Filled triangles, diamonds, and squares indicate the Néel temperature *T*_N_, structural phase transition temperature *T*_S_, and superconducting transition temperature *T*_c_ of bulk samples, respectively, as reported in Refs. [25, 42]. Filled circles mark $T_{c}^{\mathrm{onset}}$ for epitaxial thin films prepared by the same technique [27, 44]. Red and blue stars represent $T_{c}^{\mathrm{onset}}$ of samples #1 and #2, respectively. Two stars for sample #1 mark the temperature at which a multi‐step drop appears in the transient region of the *ρ*–*T* curve.

Consider a spatial distribution *n*(**r**) of dopant or constituent species with a finite gradient **∇** *n*. Treating the plane perpendicular to the gradient as uniform (neglecting in‐plane rotational anisotropy), the characters (+1 for even, −1 for odd) of **∇** *n* under the symmetry operations {2_∥_, 2_⊥_, *m*_⊥_, *m*_∥_} are {+1, −1, −1, +1}, identical to those of the electric polarization ***P*** (Figure 1a). In other words, a system endowed with a concentration gradient **∇** *n* behaves as a polar system with ***P*** || **∇** *n*. Such gradients arise naturally and ubiquitously in solids during processes involving ionic diffusion, notably topotactic reactions, and are common rather than exceptional [16, 17, 18, 19, 20, 21, 22]. Although the resulting state is formally nonequilibrium, diffusion barriers can render its relaxation exceedingly slow; it often exists as a glass‐like quasi‐stable state with long lifetime on experimental timescales. Therefore, the polar state is long‐lived. Crucially, this approach is independent of the parent crystal symmetry: even centrosymmetric hosts can be rendered polar via **∇** *n*. Moreover, when **∇** *n* is produced topotactically, the magnitude of **∇** *n* (hence the degree of P‐breaking) can be tuned continuously by electrochemical and thermal annealing.

### 2.2 Topotactically Hydrogenated SmFeAsO as a Test Platform

To demonstrate this concept, we chose SmFeAsO (Sm1111) as a test platform. As shown in Figure 1b, Sm1111 comprises alternating SmO and FeAs layers in a centrosymmetric crystal structure (space group *Cmma* at temperatures below 130 K) [23]. The undoped parent phase is a metallic antiferromagnet, while electron doping by substituting F^−^ or H^−^ for the O^2−^ site induces superconductivity [24, 25]. Sm1111 exhibits the highest superconducting transition temperature among the 1111 family of iron‐based superconductors (*T*_c_ ≈ 55 K for bulk samples [26]). Notably, epitaxial films can be electron‐doped by topotactic hydrogen substitution: Sm1111 epitaxially grown on an MgO substrate and annealed in CaH_2_ powder undergoes O^2−^ ↔ H^−^ exchange, enabling wide control of the H content (from a few to several tens of percent) and *T*_c_ (up to ∼ 48 K) [27]. Under appropriate conditions, a through‐thickness H^−^ concentration gradient emerges naturally. In such films, the host crystal lattice remains centrosymmetric, yet **∇** *n*_H_ defines a polar axis and breaks P, making Sm1111:H an ideal testbed for our strategy.

### 2.3 Nonreciprocal Charge Transport as a Probe of Inversion‐Symmetry Breaking

To detect P‐breaking, we employ nonreciprocal charge transport, a directional dichroism in charge transport that strictly requires broken P [8, 28, 29]. In polar systems, the electrical voltage drop is phenomenologically described as a function of the magnetic field *B* and the current *I* by

(1) $$V=R_{0}I\left(1+\beta B^{2}+\gamma \left(B\times \hat{z}\right)\cdot I\right)$$

where $\hat{z}$ denotes the unit vector along the polar axis [29, 30, 31, 32]. The second term represents ordinary magnetoresistance. The third term depends on the current direction and encodes nonreciprocal transport. If P is preserved, the symmetry forbids this term; therefore, observing nonreciprocal transport serves as an electrical signature of P‐breaking. Practically, since the third term appears in the measured voltage as a nonlinear response proportional to *I* ^2^, the nonreciprocal coefficient *γ* is extracted by using the second‐harmonic resistance *R* ^2ω^ = γ*R*_0_ *BI*/2 under ac current and in‐plane magnetic fields, most sensitively for ***I*** ⊥ ***B*** (see Note S1). Moreover, in superconductors, the nonreciprocity is strongly amplified near the superconducting transition via superconducting fluctuations (paraconductivity) [33, 34, 35] and vortex dynamics [34, 36, 37, 38, 39]. The Sm1111 epitaxial thin film with a hydrogen gradient **∇** *n*_H_ is therefore ideally suited to demonstrate concentration‐gradient‐induced P‐breaking through the detection of nonreciprocal charge transport (Figure 1c).

## 3 Results and Discussion

### 3.1 Sample Characterization

Sm1111:H epitaxial thin films were prepared following procedures established in previous reports [27, 40]. The *c*‐axis–oriented undoped Sm1111 films were first grown on MgO(001) substrates by pulsed laser deposition (PLD), after which oxygen was partially replaced by hydrogen via topotactic post‐annealing in CaH_2_ powder. In this study, we fabricated two films with distinct hydrogen distributions: sample #1 (thickness 60 nm) and sample #2 (68 nm). Figure 1d shows the depth profiles of hydrogen concentration *x* measured by secondary‐ion mass spectrometry (SIMS). In sample #2, the hydrogen level is nearly uniform throughout the thickness, with an average *x* ≈ 0.04. In contrast, sample #1 exhibits a pronounced through‐thickness gradient: *x* ≈ 0.14 near the top surface, decreasing to *x* ≈ 0.04 close to the Sm1111/MgO interface. Note that the apparent spike at the surface and the perturbation near the interface, where the composition changes abruptly, are not intrinsic; they are attributable to a well‐known SIMS pile‐up effect [41]. Additionally, the vicinity of the MgO substrate is susceptible to charging because of its highly insulating nature. Although H doping up to ∼ 35% and a maximum *T*_c_ of ∼ 48 K are achievable under optimized annealing conditions [27], the present study deliberately employed lightly doped films to facilitate unambiguous verification of the H concentration gradient via transport measurements. Because the *T*_c_–*x* phase diagram of Sm1111:H is dome‐shaped [25, 42], *T*_c_ varies little with *x* near the dome center; thus, the H gradient has little apparent effect on the behavior of the superconducting transition in this regime. In contrast, on the underdoped side (the dome edge), *T*_c_ depends sensitively on *x*, so the H concentration gradient is expected to manifest as a multi‐step superconducting transition. Figure 1e displays the temperature dependence of the electrical resistivity *ρ*(*T*) for sample #1. Clear metallic behavior is observed in the normal state, and a drop of *ρ* indicating a superconducting transition, appears with $T_{c}^{\mathrm{onset}}$ = 41 K. Although applying a magnetic field perpendicular to the *c* axis shifts *T*_c_ slightly to lower temperatures, superconductivity persists up to 9 T, reflecting an extremely high upper critical field and reproducing earlier reports [27, 43, 44]. The inset of Figure 1e compares *ρ*(*T*) for samples #1 and #2 on a logarithmic scale. At first glance, the two films appear to display similar *ρ*–*T* curves with nearly identical *T*_c_ values (see Note S4). The uniform film (sample #2) shows a sharp fall near $T_{c}^{\mathrm{onset}}$ = 41.5 K, whereas the gradient film (sample #1) exhibits a multi‐step decrease with shoulder‐like structures around the transition. Such multi‐step behavior is characteristic of disordered superconductors that can be described as superconducting islands embedded in a normal‐metal matrix [45, 46, 47, 48]. In sample #1, the hydrogen gradient along the film‐thickness direction leads to a distribution of local *T*_c_ values, with regions near the surface tending to exhibit higher *T*_c_ values. If additional in‐plane inhomogeneity is present, the formation of superconducting islands and the global superconducting transition, where the inter‐islands percolate, occur at distinct temperatures. As a result, the superconducting transition is expected to appear as a multi‐step feature in transport measurements. These observations support the presence of strong disorder induced by the formation of a hydrogen concentration gradient within the film. As summarized in Figure 1f, the *T*_c_ values extracted from the *ρ*–*T* curves are plotted on the established phase diagram. While the *T*_c_ values are slightly reduced compared with those of bulk samples, the $T_{c}^{\mathrm{onset}}$ values of samples #1 and #2 track the superconducting dome reasonably well.

### 3.2 Nonreciprocal Charge Transport in Sm1111 with **∇** *n*_**H**_

**FIGURE 2:**
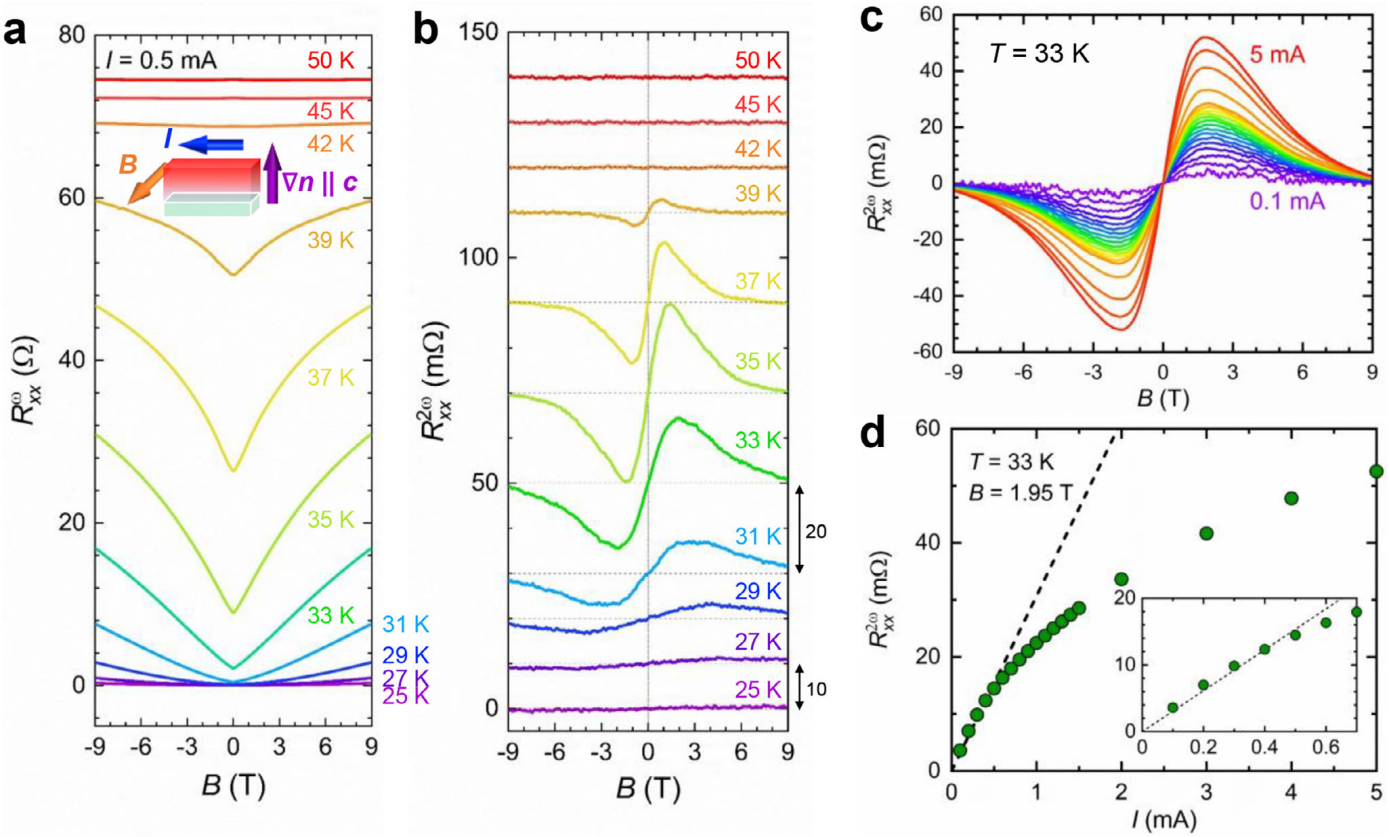
Temperature and current‐amplitude dependences of second‐harmonic magnetoresistance in Sm1111 with **∇** *n*_H_. (a, b) Magnetic‐field dependence of the first‐ and second‐harmonic resistance ($R_{xx}^{\omega }$ and $R_{xx}^{2\omega }$, respectively) of sample #1 for various temperatures across the superconducting transition ($T_{c}^{\mathrm{onset}}$ = 41 K). As shown in the inset schematic, the magnetic field and electric current are applied mutually orthogonally, and both are perpendicular to the hydrogen concentration gradient. The amplitude of the input AC current was 0.5 mA. For clarity, the curves in (b) are vertically offset by 10 or 20 mΩ. (c) Magnetic‐field dependence of $R_{xx}^{2\omega }$ at *T* = 33 K under various current amplitudes ranging from 0.1 to 5 mA. The magnetic field and current geometries are identical to those in the inset of Figure 2a. (d) Current‐amplitude dependence of $R_{xx}^{2\omega }$ under *B* = 1.95 T at *T* = 33 K. The dashed line is a guide to the eye, indicating the linear relationship between $R_{xx}^{2\omega }$ and current amplitude at the low‐current regime. The inset shows an enlarged view of the low‐current regime.

We next focus on the charge transport properties under ac excitation. Gold electrodes for conventional four‐terminal measurements were deposited on the prepared films by sputtering, and Au wires were bound using silver paste; no special nanofabrication, such as focused ion beam (FIB) processing or electrode patterning, was employed. Unless otherwise noted, we applied an ac current of *I*_ac_ = 0.5 mA (current density *j*_ac_ ≈ 10^2^ A/cm^2^) and measured the first‐ and second‐harmonic resistances, $R_{\mathrm{xx}\omega }$ and $R_{xx}^{2\omega }$, using a lock‐in amplifier. Figure 2a,b shows the magnetic‐field dependence of $R_{xx}^{\omega }$ and $R_{xx}^{2\omega }$, respectively, for sample #1 at various temperatures across *T*_c_. The magnetic field ***B*** and current ***I*** were applied mutually orthogonally and both perpendicular to the hydrogen concentration gradient, that is, ***B*** ⊥ ***I*** ⊥**∇** *n*_H_ (see inset schematic). In the normal state well above *T*_c_, changes in both $R_{xx}^{\omega }$ and $R_{xx}^{2\omega }$ were too small to be detected by our experimental setup. Upon approaching the transient regime, $R_{xx}^{\omega }$ displays a large positive magnetoresistance, reflecting the *B*‐induced suppression of superconductivity. At the same temperature, $R_{xx}^{2\omega }$ becomes finite and exhibits an antisymmetric peak‐valley structure centered at *B* = 0 T. This line shape is the canonical fingerprint of nonreciprocal charge transport in superconductors, as reported previously [33, 34, 49]. The observation of the finite $R_{xx}^{2\omega }$ thus indicates that sample #1 is characterized by broken P. Figure 2c presents $R_{xx}^{2\omega }$–*B* curves at 33 K, where the signal is the most pronounced, for various current amplitudes. The antisymmetric structure grows monotonically with increasing *I*. The current‐amplitude dependence of $R_{xx}^{2\omega }$ at the peak‐valley field (*B* = 1.95 T) is summarized in Figure 2d in the low‐current regime (≲ 0.5 mA; inset), $R_{xx}^{2\omega }$ increases linearly with *I*_ac_, while at higher currents it deviates from the linear relationship, plausibly due to Joule heating, current‐induced suppression of superconductivity, and/or higher‐order effect. These results demonstrate that $R_{xx}^{2\omega }\propto$ *BI*, that is, the expected scaling for nonreciprocal transport, holds in the low‐magnetic‐field and low‐current limit. Furthermore, the observation that $R_{xx}^{2\omega }$ appears exclusively in the vicinity of the superconducting transition and remains absent in the normal state clearly rules out thermoelectric artifacts such as the Nernst effect caused by Joule heating at a current contact [50, 51]. This finding indicates that the nonreciprocal charge transport observed in sample #1 is intimately associated with the superconducting transition.

### 3.3 Geometry Dependence of Nonreciprocal Charge Transport

**FIGURE 3:**
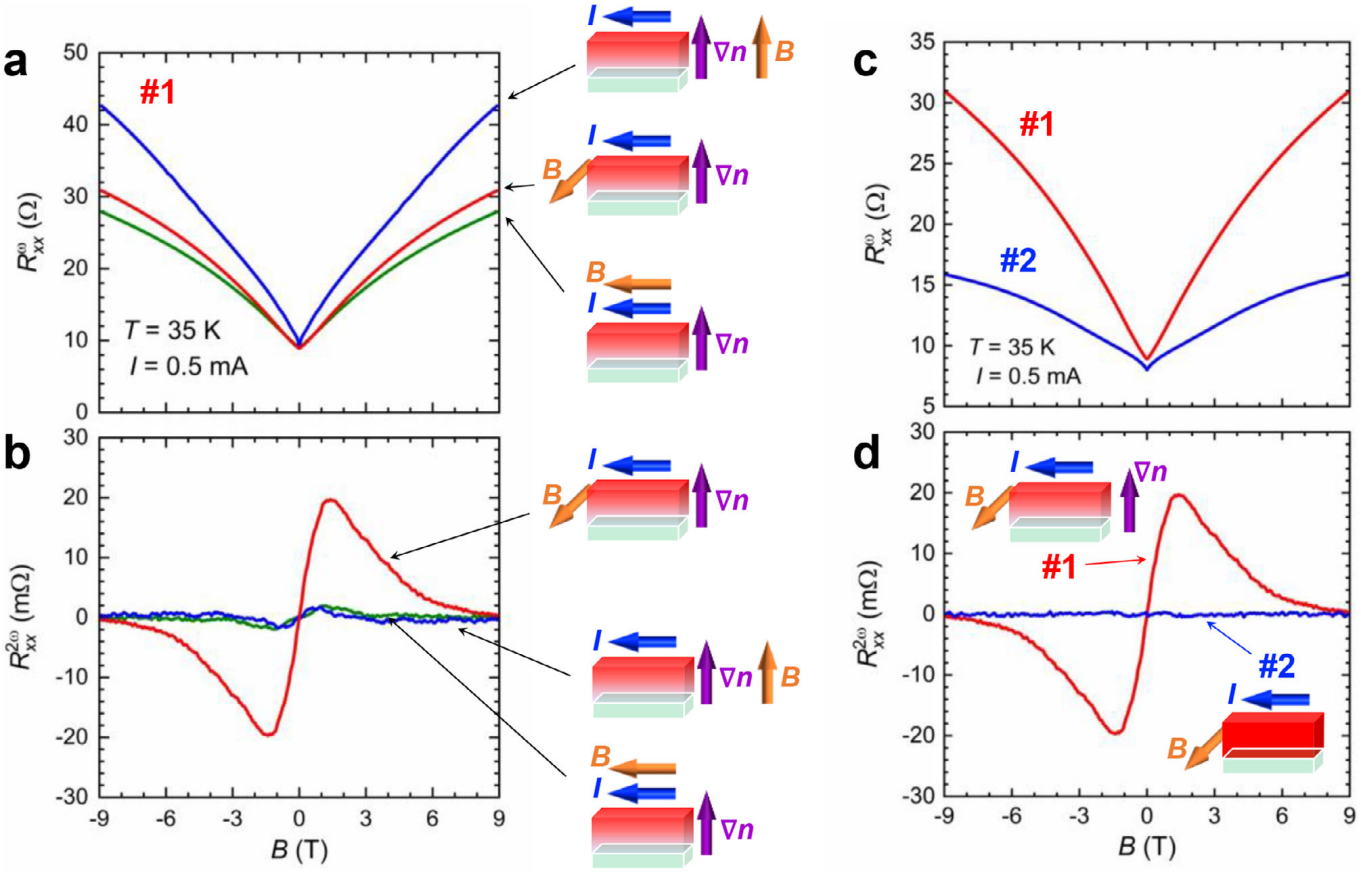
Geometry and concentration‐gradient dependences of nonreciprocal charge transport. (a, b) Magnetic‐field dependence of $R_{xx}^{\omega }$ and $R_{xx}^{2\omega }$ of sample #1 at *T* = 33 K with *I*_ac_ = 0.5 mA, measured under various magnetic‐field and current configurations: (i) ***I*** ⊥ ***B*** ⊥ **∇** *n*_H_, (ii) ***I*** ⊥ ***B*** || **∇** *n*_H_, (iii) ***I*** || ***B*** ⊥ **∇** *n*_H_. (c, d) Comparison of the magnetic‐field dependences of the $R_{xx}^{\omega }$ and $R_{xx}^{2\omega }$ between sample #1 (with **∇** *n*_H_) and sample #2 (without **∇** *n*_H_). The magnetic field and current were arranged in configuration (i) ***I*** ⊥ ***B*** ⊥ **∇** *n*_H_ with *I*_ac_ = 0.5 mA at *T* = 35 K.

To identify the origin of P‐breaking, we measured $R_{xx}^{\omega }$ and $R_{xx}^{2\omega }$ under several current and magnetic field geometries. Figure 3a,b shows the magnetic field dependence of $R_{xx}^{\omega }$ and $R_{xx}^{2\omega }$, respectively, for three configurations: (i) ***I*** ⊥ ***B*** ⊥ **∇** *n*_H_, (ii) ***I*** ⊥ ***B*** || **∇** *n*_H_, and (iii) ***I*** || ***B*** ⊥ **∇** *n*_H_. Although the amplitudes differ, $R_{xx}^{\omega }$ shows a positive magnetoresistance in all the three geometries. In contrast, a pronounced antisymmetric peak‐valley structure in $R_{xx}^{2\omega }$ appeared only for geometry (i). Weak residual $R_{xx}^{2\omega }$ signals in the other geometries may be attributed to slight misalignment. Indeed, the high accuracy of the experimental configuration is evidenced by the excellent reproducibility observed upon repeated remounting of the sample (see Note S5). This clear geometry selectivity accords with the selection rules of Equation (1): with the polar axis *z* || **∇** *n*_H_, the nonreciprocal term γ(***B*** × ***z***) · ***I*** is maximized when ***I*** || (***B*** × ***z***) (i.e., ***I*** ⊥ ***B*** ⊥ ***z***) and vanishes for ***B*** || ***z*** or ***I*** || ***B***. These results indicate that sample #1 realizes a polar P‐broken state that is symmetry equivalent to an electric polarization ***P*** aligned with **∇** *n*_H_. Moreover, if an in‐plane concentration gradient $\nabla n_{H}^{\mathrm{in}-\mathrm{plane}}$ were present, the configuration ***B*** || ***z*** would still satisfy the condition ***I*** ⊥ ***B*** ⊥ $\nabla n_{H}^{\mathrm{in}-\mathrm{plane}}$, under which the nonreciprocal electrical transport is expected to be observable. The absence of such a nonreciprocal response therefore clearly rules out the presence of an in‐plane concentration gradient.

### 3.4 Selective Emergence of Nonreciprocity in Gradient‐Engineered Films

Figure 3c,d compare $R_{xx}^{\omega }$ and $R_{xx}^{2\omega }$ for the two films in a fixed geometry, where ***B***, ***I*** and the film normal (thickness direction) are mutually orthogonal (see inset figures). Both samples exhibit positive magnetoresistance in $R_{xx}^{\omega }$. However, a robust $R_{xx}^{2\omega }$ signal is observed only in sample #1 (with a hydrogen concentration gradient), whereas no $R_{xx}^{2\omega }$ is detected in sample #2 (without a gradient). This contrast demonstrates that the nonreciprocal charge transport originates from **∇** *n*_H_. Strictly speaking, the surface and the Sm1111/MgO interface can also locally break inversion symmetry. Furthermore, contributions from epitaxial strain arising from the lattice mismatch between the substrate and the film may be present. Nevertheless, the absence of the finite $R_{xx}^{2\omega }$ signal in sample #2 excludes these effects as dominant sources. The observed nonreciprocity is therefore governed primarily by the hydrogen concentration gradient.

### 3.5 Origin of Nonreciprocal Charge Transport in Sm1111 with ∇*n*_**H**_

**FIGURE 4:**
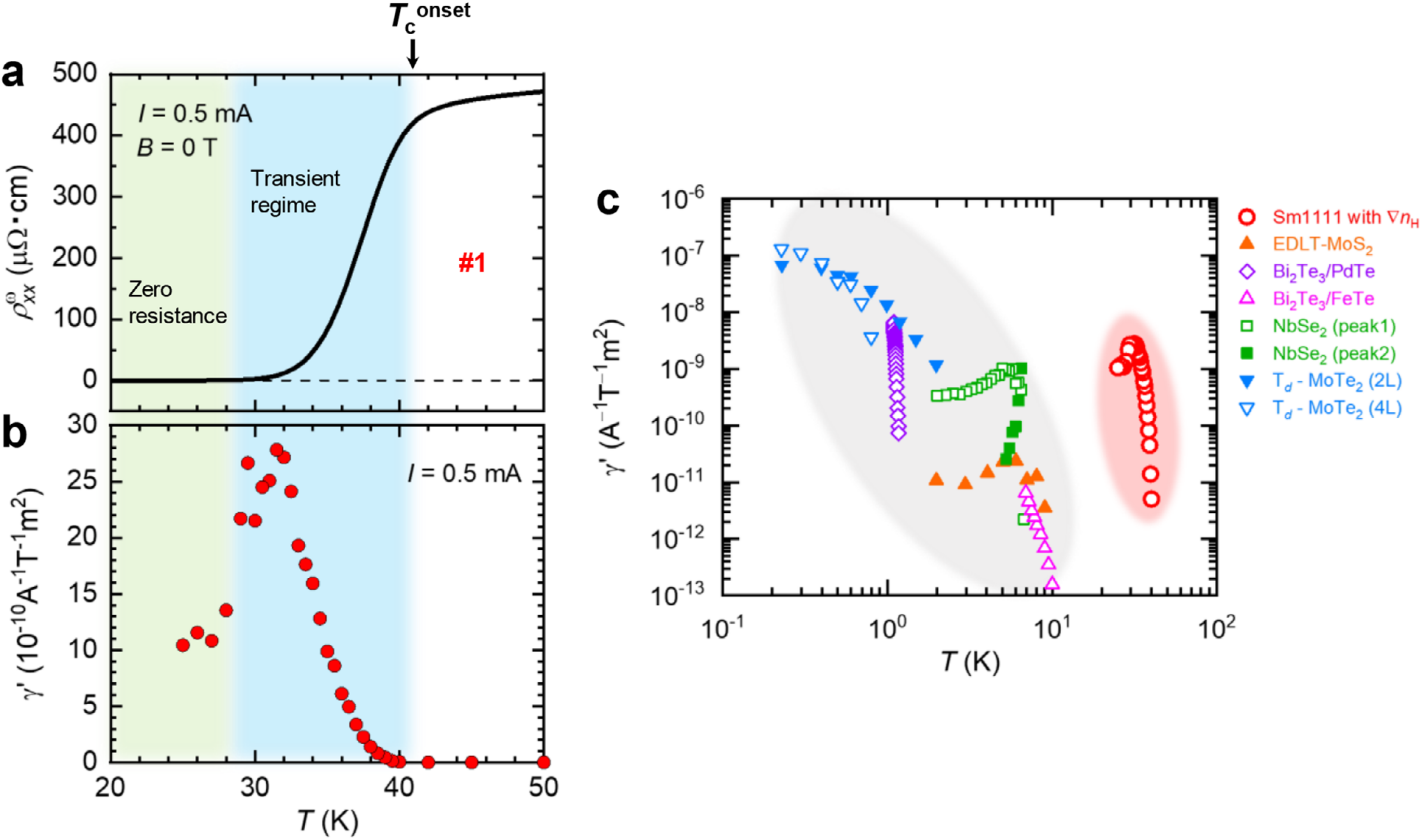
Temperature evolution of the nonreciprocal coefficient and comparison across systems. (a, b) Temperature dependence of the first‐harmonic resistivity $\rho_{xx}^{\omega }$ and the nonreciprocal coefficient γ′ of sample #1 under AC current amplitude of *I*_ac_ = 0.5 mA. We adopt a size‐independent nonreciprocal coefficient, γ′ = γ/*S*, where *S* is the cross‐sectional area. Here, $\gamma =\frac{2R_{xx}^{2\omega }}{R_{xx}^{\omega }BI_{\mathrm{ac}}}$ is evaluated from the slope of the $R_{xx}^{2\omega }/R_{xx}^{\omega }$–*B* profile near 0 T, derived from Figure 2a,b. (see Note S2). The green shading roughly denotes the fully superconducting state with zero resistance, as a guide to the eye. The blue shading indicates the transient regime between $T_{c}^{\mathrm{onset}}$ (the onset of the resistivity drop) and the zero‐resistance temperature. (c) Size‐independent nonreciprocal coefficient γ′ as a function of temperature for superconductors exhibiting vortex‐motion‐driven nonreciprocal charge transport [33, 36, 38, 39, 65]. The data for Sm1111 with **∇** *n*_H_ studied here are marked by open red circles. To estimate the value of γ′ for interfacial 2D superconductors EDLT‐MoS_2_ [33], Bi_2_Te_3_/PdTe [39], and Bi_2_Te_3_/FeTe [36], we adopt an effective superconducting thickness of 1 nm, following Ref. [49].

To address the mechanism of the nonreciprocal charge transport in sample #1, we qualitatively evaluate the nonreciprocal coefficient γ. Several definitions exist in the literature [34, 36, 52]; here we adopt the form derived from Equation (1), $\gamma =\frac{2R_{xx}^{2\omega }}{R_{xx}^{\omega }BI_{0}}$, which is evaluated from the slope of $R_{xx}^{2\omega }/R_{xx}^{\omega }$–*B* curve around 0 T following previous studies [36, 49] (see Note S2). In superconductors, there are two principal mechanisms for nonreciprocal charge transport, particularly formulated for 2D superconductors: (I) nonreciprocal paraconductivity governed by amplitude fluctuations of the superconducting order parameter [8, 33, 35], and (II) vortex‐motion‐driven nonreciprocity [34, 36, 37, 38, 39, 52]. In scenario (I), γ increases upon approaching the mean‐field transition temperature *T*_c0_ and is maximal on the higher‐temperature side of the transient regime. In scenario (II), γ grows toward the Berezinskii–Kosterlitz–Thouless (BKT) temperature *T*_BKT_, where vortices and antivortices bind into pairs, and is maximal on the lower‐temperature side of the transient regime. Our Sm1111:H films are bulk superconductors with a thickness *d* ≈ 50 nm. The reported coherence lengths *ξ_c_* of Sm1111:H epitaxial thin films are a few nanometers [27]. Therefore, *d* ≫ *ξ_c_* and the order parameter is effectively 3D. Consequently, order parameter fluctuations are suppressed, and a dominant contribution from paraconductivity is unlikely. Although the paraconductivity is negligible owing to the 3D nature, an applied magnetic field readily creates vortex lines, and vortex motion can provide an active route to nonreciprocity. Figure 4a,b summarizes the temperature dependences of $\rho_{xx}^{\omega }$ and γ′, where is the size‐independent coefficient defined as γ/*S* (*S*: cross sectional area). The γ′ begins to develop at $T_{c}^{\mathrm{onset}}$ and then grows steeply toward lower temperatures within the transient regime. This trend favours vortex motion over paraconductivity as the primary origin of the nonreciprocal response. A microscopic picture is as follows. An in‐plane magnetic field nucleates vortex lines (for example, the lower critical field *H*_c1_ in polycrystalline SmFeAsO_1−_ *_x_* F*_x_* is ∼13 mT [53], and the in‐plane *H*_c1_ of single crystalline SmFe_0.92_Co_0.08_AsO is ∼4 mT [54], indicating that vortices are easily generated.). It is also noted that a Sm1111:H epitaxial film shows a small anisotropy of the upper critical field (*H*_*c*2||_/*H*_*c*2⊥_ ≈ 2) [43]. Moreover, the coherence length along the *c* axis *ξ_c_* at 0 K is estimated to be approximately 13 nm, which is sufficiently longer than the *c*‐axis lattice constant (∼ 0.85 nm) [43]. These findings indicate 3D vortex dynamics and suggest the formation of Abrikosov vortices rather than Josephson vortices for in‐plane fields [55]. When an in‐plane current perpendicular to the field is applied, the Lorentz force drives vortex motion along the film normal. The hydrogen concentration gradient produces an asymmetric potential landscape along **∇** *n*_H_, potentially originating from gradients in the lattice constant and/or the density of pinning centers. This asymmetry leads to the directional dependence of vortex mobility along the film normal (|| **∇** *n*_H_), giving rise to a finite second‐harmonic voltage and manifesting as nonreciprocity, i.e., *R*(*I*, *B*) ≠ *R*(−*I*, *B*) and/or *R*(*I*, *B*) ≠ *R*(*I*, −*B*). Nonreciprocal charge transport arising from an imbalance of vortex motion has been observed in other asymmetric systems [38, 56].

Figure 4c compares the temperature evolution of γ′ in gradient‐engineered Sm1111 with that in superconductors exhibiting nonreciprocal charge transport arising from vortex motion. In prior materials, the relatively low *T*_c_ (typically < 10 K) confines detectable γ′ to cryogenic temperatures, and γ′ generally grows upon cooling. In contrast, the high‐*T*_c_ Sm1111 with **∇** *n*_H_ exhibits nonreciprocity already from ∼40 K, with comparatively large γ′, markedly deviating from the established trend. Notably, Sm1111:H shows the highest onset temperature of vortex‐motion induced nonreciprocity characterized by γ′ reported to date. These findings indicate that concentration‐gradient engineering offers a practical route to P‐breaking that produces robust nonreciprocal responses at elevated temperatures.

As an extreme manifestation of nonreciprocal transport in superconductors, the superconducting diode effect (SDE)—in which dissipationless current flows in one direction while a finite resistance appears in the opposite direction—has attracted considerable attention in recent years [8, 57]. Notably, SDE has been observed at temperatures exceeding ∼40 K, higher than those accessible in the present system, in high‐*T*_c_ cuprate superconductors, particularly in exfoliated flakes [58] and in artificially layered structures [59, 60]. Although a perfect SDE is not realized in Sm1111:H studied here, the present results suggest that the introduction of P‐breaking via a concentration gradient provides a general route to inducing SDE not only in this system but also in a wide range of superconductors.

## 4 Conclusions

In conclusion, we have proposed a symmetry‐based strategy in which a concentration gradient **∇** *n* can serve as a new platform for realizing an inversion symmetry (P)‐broken state. We have demonstrated this concept by observing the benchmark signature of P‐breaking—nonreciprocal charge transport—in SmFeAsO (Sm1111) epitaxial thin films with a hydrogen concentration gradient **∇** *n*_H_. From the geometry selectivity of the second‐harmonic response and the γ–*T* curve, we conclude that the dominant origin of the nonreciprocal charge transport in Sm1111 with **∇** *n*_H_ is vortex‐motion nonreciprocity arising from an asymmetric pinning potential landscape created by **∇** *n*_H_. We further note that, unlike conventional Sm1111, P‐breaking introduced by **∇** *n*_H_ might allow a unique superconducting state typified by the singlet‐triplet mixing, as discussed in non‐centrosymmetric superconductors [6, 61].

In the present study, the concentration gradient was realized via a topotactic reaction that introduced an asymmetric dopant profile. Since topotactic ion‐exchange routes, including isotope substitution [62] and electrochemical insertion/extraction [63], are well established in solid‐state science, our approach is broadly applicable to a wide range of material systems. While our focus was on nonreciprocal transport in superconductors, the same symmetry principle should enable optical second‐harmonic generation, piezoelectric responses, and other inversion‐symmetry‐odd phenomena in gradient‐engineered platforms. More broadly, concentration‐gradient engineering offers a versatile and chemically general route to creating P‐broken states, opening a pathway to a wider landscape of functional materials.

## 5 Experimental Section

### 5.1 Sample Preparation

The undoped Sm1111 heteroepitaxial thin films were grown on (001)‐oriented MgO single‐crystalline substrates (size: 10 mm × 10 mm × 0.5 mm) by PLD using the second harmonic (𝜆 = 532 nm) of an Nd:YAG laser. The Nd:YAG PLD‐film‐growth procedure followed the details reported in Ref. [40]. Hydrogen substitution in Sm1111 was achieved by topotactic ion exchange between the as‐grown epitaxial Sm1111 films and CaH_2_ powder. Further procedural details are provided in Ref. [27]. Depth profiles of the hydrogen concentration were obtained by SIMS at Toray Research Center, Inc.

### 5.2 Charge Transport Measurements

After the annealing procedure, the film‐grown substrates were diced into bar‐shaped specimens with dimensions of 10 mm × 2 mm × 0.5 mm. Au electrodes for four‐probe measurements were deposited, and Au wires were attached with conductive Ag paste using standard procedures. The spacing between the voltage contacts is approximately 1.5 mm. Notably, no micro‐/nanofabrication, such as FIB processing, was employed for electrode patterning or wiring. The first‐ and second‐harmonic resistances, *R^ω^* and *R* ^2^ *^ω^*, were measured using an ac current source (Model 6221, Keithley) and lock‐in amplifiers (LI5640, NF Corporation). Unless otherwise noted, the amplitude and frequency of the injected current were set to 0.5 mA (root mean square) and 13 Hz, respectively. All transport measurements were performed using a Physical Property Measurement System (PPMS; Quantum Design). During the ac resistance measurements, the phases of the first‐ and second‐harmonic signals were confirmed to be approximately 0 and π/2, respectively, consistent with the theoretical expectation (see Note S1). All *R^ω^* (or *R* ^2^ *^ω^*) signals presented in the main text correspond to the *x* (or *y*) components of the lock‐in output, respectively. The *R^ω^* and *R* ^2^ *^ω^* data were symmetrised and antisymmetrised, respectively, with respect to the magnetic field *B* (see Note S3).

## Funding

KAKENHI (Grant Nos. JP24K08561, JP 25H01247, and JP 25H00392), Murata Science and Education Foundation (No. M25AN162), Ministry of Education, Culture, Sports, Science, and Technology (MEXT) through the Element Strategy Initiative to Form Core Research Center (Grant No. JPMXP0112101001)

## Conflicts of Interest

The authors declare no conflicts of interest.

## Supporting information




**Supporting File**: advs74312‐sup‐0001‐SuppMat.docx.

## Data Availability

The data that support the findings of this study are available from the corresponding author upon reasonable request.
