# Supplementary material for: Nonreciprocal Charge Transport in an Iron‐Based Superconductor with Broken Inversion Symmetry Engineered by a Hydrogen‐Concentration Gradient
Source: Adv Sci (Weinh). 2026 Feb 15;13(22):e24270. doi: 10.1002/advs.202524270 (PMC13088315; doi:10.1002/advs.202524270)
Supplement: Supplementary file 1 — Supporting File: advs74312‐sup‐0001‐SuppMat.docx. [file ADVS-13-e24270-s001.docx]

Supporting Information

Nonreciprocal Charge Transport in an Iron-Based Superconductor with Broken Inversion Symmetry Engineered by a Hydrogen-Concentration Gradient

Takayuki Nagai*, Yukito Nishio, Jumpei Matsumoto, Kota Hanzawa, Hidenori Hiramatsu, Hideo Hosono, and Tsuyoshi Kimura

**Supplementary Note 1 | Nonreciprocal charge transport measurement**

Phenomenologically, the electrical resistance can be expanded up to second order in magnetic field and current as

$$\begin{aligned} R\left( I, B \right)=R_{0}\left( 1+\beta B^{2}+\gamma BI \right),\#\left( S1 \right) \end{aligned}$$

where *R*_0_, *I*, and *B* are the zero-field resistance, the applied current, and the magnetic field, respectively.^[S1, S2]^ The second term with coefficient *β* represents ordinary magnetoresistance. The third term depends on the directions of *I* and *B* and produces a nonreciprocal component, as seen from $\Delta R=R\left( B, I \right)-R\left( B, -I \right)\propto\gamma BI$, which is odd under reversal of *I*. In centrosymmetric systems, this third term is forbidden by symmetry, implying *γ* = 0 when inversion symmetry is preserved. Observation of a finite nonreciprocal response therefore provides an electrical probe of inversion symmetry breaking.

　For a polar system, symmetry refines Eq. (S1) to

$$\begin{aligned} R\left( I, B \right)=R_{0}\left[ 1+\beta B^{2}+\gamma\left( \boldsymbol{B}\times\hat{\boldsymbol{z}} \right)\cdot\boldsymbol{I} \right],\#\left( S2 \right) \end{aligned}$$

where $\hat{\boldsymbol{z}}$ is the unit vector along the polar axis. The current-dependent resistance generates a nonlinear voltage drop that can be detected by second-harmonic lock-in techniques under an ac current $I\left( t \right)=I_{\mathrm{ac}}\sin\omega t$.^[S3]^ The voltage originating from the third term yields

$$V^{2\omega}\left( t \right)=\gamma R_{0}BI_{\mathrm{ac}}^{2}\cos\theta\sin^{2} \omega t$$

$$\begin{aligned} =\frac{1}{2}\gamma R_{0}BI_{\mathrm{ac}}^{2}\cos\theta\left\{ 1+\sin\left( 2\omega t-\frac{\pi}{2} \right) \right\},\#\left( S3 \right) \end{aligned}$$

where *θ* is the angle between ***B*** and ***I***. Consequently, in the low-field limit, the first- and second-harmonic resistances become

$$R^{\omega}\equiv\frac{V^{\omega}}{I_{\mathrm{ac}}}\approx R_{0}, R^{2\omega}\equiv\frac{V^{2\omega}}{I_{\mathrm{ac}}}=\frac{1}{2}\gamma R_{0}BI_{\mathrm{ac}}\cos\theta.$$

In our experiments we detected the lock-in *y*-component of the second-harmonic voltage with a π/2 phase shift, and confirmed that the *x*-component was almost zero.

**Supplementary Note 2 | Evaluation of the nonreciprocal coefficient *γ***

　For the orthogonal geometry with $\hat{\boldsymbol{z}}\perp\boldsymbol{B}\perp\boldsymbol{I}$, Eq. (S2) gives

$$\frac{R^{2\omega}}{R^{\omega}}=\frac{1}{2}\gamma I_{\mathrm{ac}}B.$$

Thus, *γ* is obtained from the slope of the low-field linear region of the ${R^{2\omega}}/{R^{\omega}}$-*B* curve. Figure S1 shows representative ${R^{2\omega}}/{R^{\omega}}$–*B* curves at several temperatures across the superconducting transition temperature *T*_c_. We evaluate *γ* by least-squares linear fitting around ***B*** = 0.

**Supplementary Note 3 | Symmetrisation and antisymmetrisation of raw data**

　To separate the even and odd components with respect to the applied magnetic field, we symmetrize and antisymmetrize the raw magnetoresistance $R_{\exp}\left( B \right)$ measured at positive and negative fields as

$$R_{\mathrm{sym}}=\frac{R_{\exp}\left( B \right)+R_{\exp}\left( -B \right)}{2}, R_{\mathrm{asym}}=\frac{R_{\exp}\left( B \right)-R_{\exp}\left( -B \right)}{2}.$$

Here, $R_{\mathrm{sym}}\left( B \right)$ is even and $R_{\mathrm{asym}}\left( B \right)$ is odd with respect to *B*. The same procedure is applied to both $R^{\omega}$ and $R^{2\omega}$ throughout the analysis. Typical raw and symmetrized/antisymmetrized datasets are shown in Figure S2. The small residual even components in $R^{2\omega}$ are likely attributable to slight contact-resistance asymmetries.

**Supplementary Note 4 | Basic transport properties of prepared samples**

　Figures S3a and 3b display the temperature dependence of the electrical resistivity for hydrogen-doped Sm1111 epitaxial thin films: sample #1 (with a concentration gradient) and sample #2 (with uniform hydrogenation). In the main manuscript, $T_{c}^{\mathrm{onset}}$ is defined as the intersection of two linear fits, one to the normal state and the other to the transient region.

**Supplementary Note 5 | Reproducibility and alignment accuracy of nonreciprocal charge transport measurements**

　Nonreciprocal charge transport is highly sensitive to the relative orientations of the applied magnetic field and electric current, and even slight misalignment in the experimental configuration can alter the magnitude of the nonreciprocal response. To assess the accuracy of our sample alignment in this study, we examined variations in the nonreciprocal charge transport upon remounting the sample in the PPMS measurement pack. As shown in Figure S4, although minor changes are observed after remounting, both the magnetic-field-odd behavior and the overall magnitude of the nonreciprocal signal are well reproduced. We therefore conclude that alignment-related errors are negligible and do not affect the discussion or conclusions of the present study.


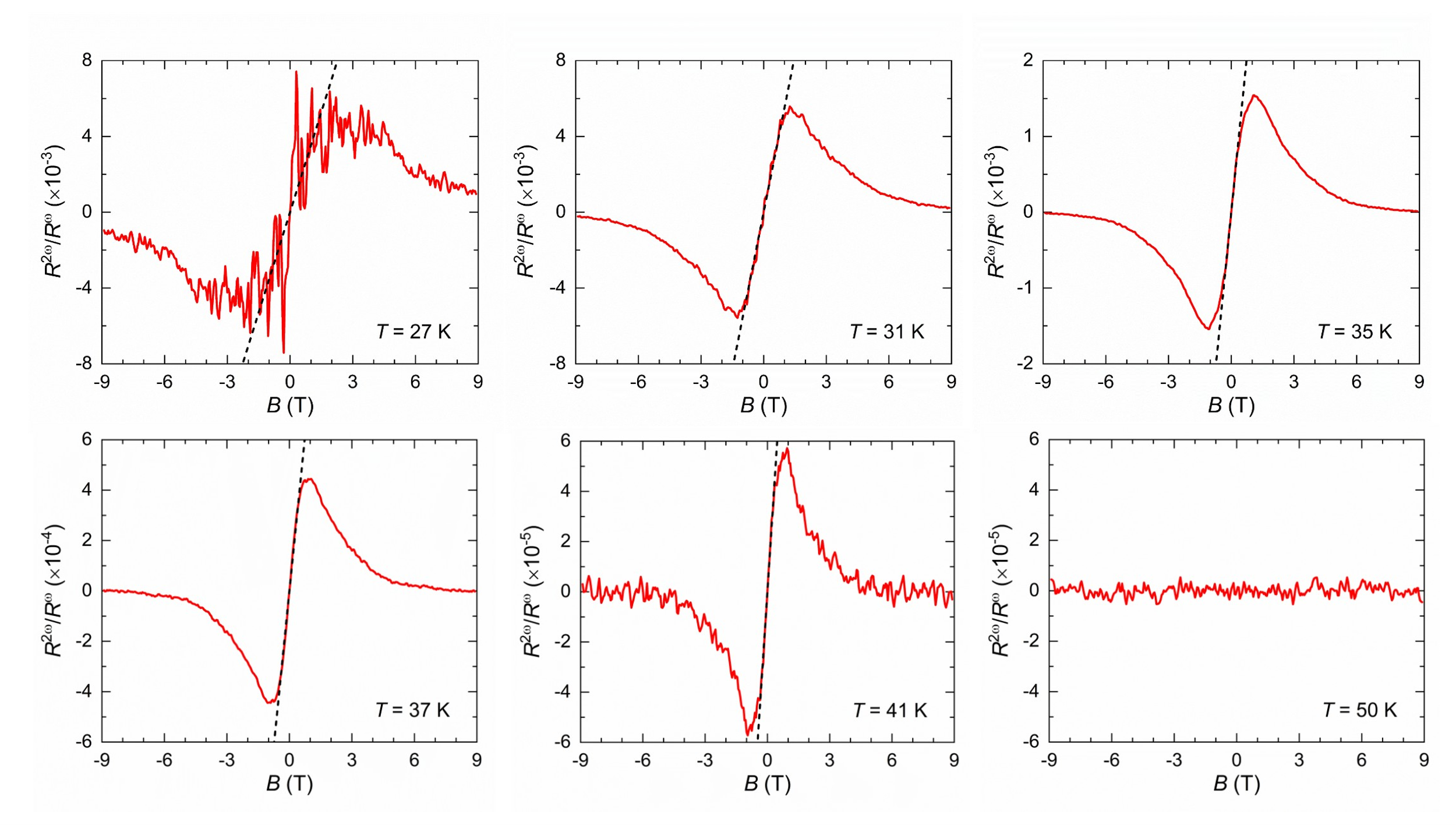


**Supplementary Figure S1 | Evaluation of the nonreciprocal coefficient *γ*.** Magnetic-field dependence of ${R_{xx}^{2\omega}}/{R_{xx}^{\omega}}$ measured under *I*_ac_ = 0.5 mA at temperatures across *T*_c_. Dashed lines indicate linear fits to the low-field regime. The nonreciprocal coefficient *γ* is obtained from the slope of the ${R_{xx}^{2\omega}}/{R_{xx}^{\omega}}$–*B* curve around 0 T. At 50 K, well above the transition temperature ($T_{c}^{\mathrm{onset}}$ = 41 K), $R_{xx}^{2\omega}$​ is effectively zero (below the detection limit); consequently, ${R_{xx}^{2\omega}}/{R_{xx}^{\omega}}$​ shows no discernible signal.


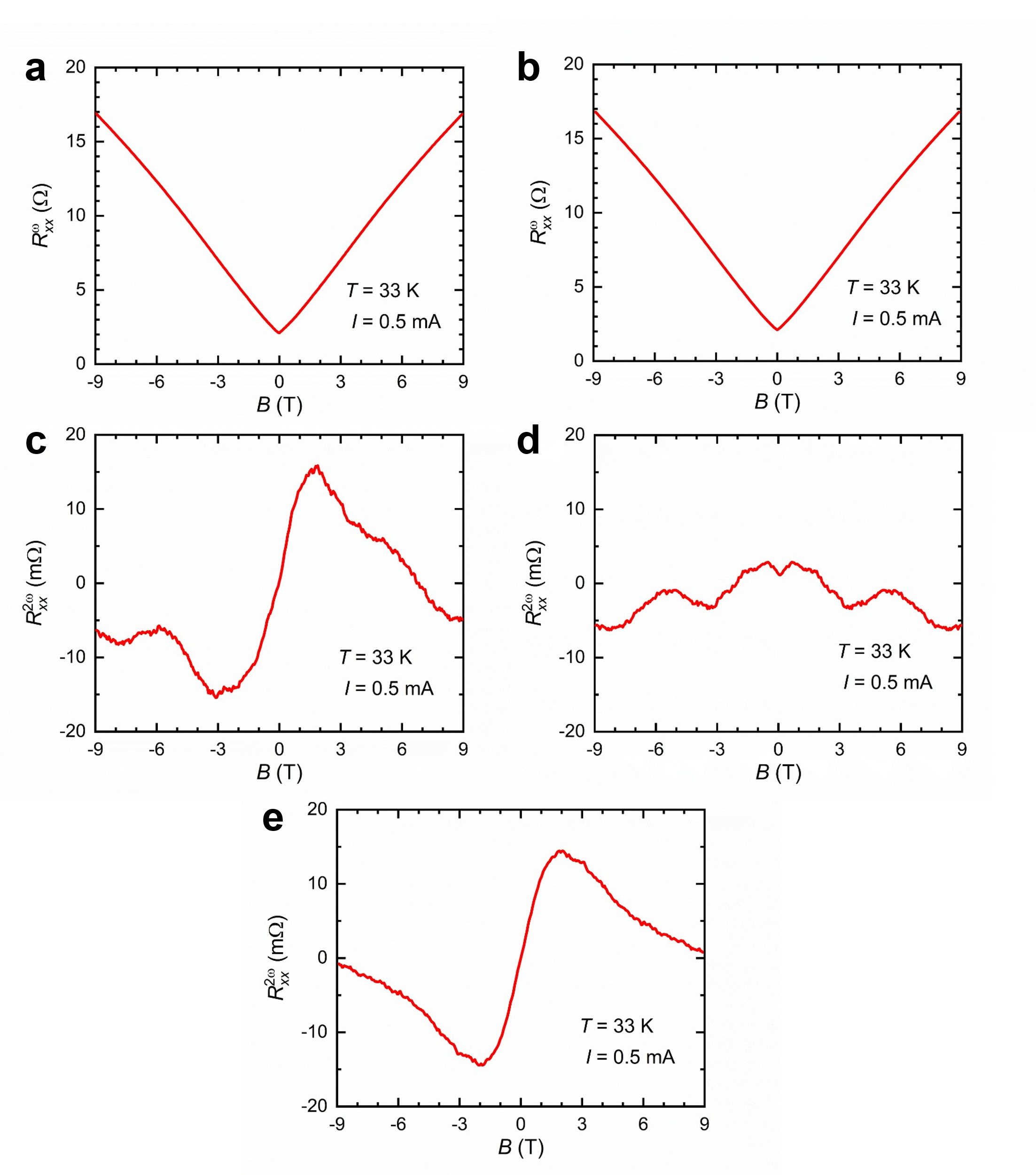


**Supplementary Figure S2 | Symmetrization and antisymmetrization of first- and second-harmonic magnetoresistance.** a, b) Raw (a) and symmetrized (b) first-harmonic magnetoresistance $R_{xx}^{\omega}$ measured under *I*_ac_ = 0.5 mA at 33 K. c, d, e) Raw (c), symmetrized (d), and antisymmetrized (e) second-harmonic magnetoresistance $R_{xx}^{2\omega}$ measured under *I*_ac_ = 0.5 mA at 33 K.


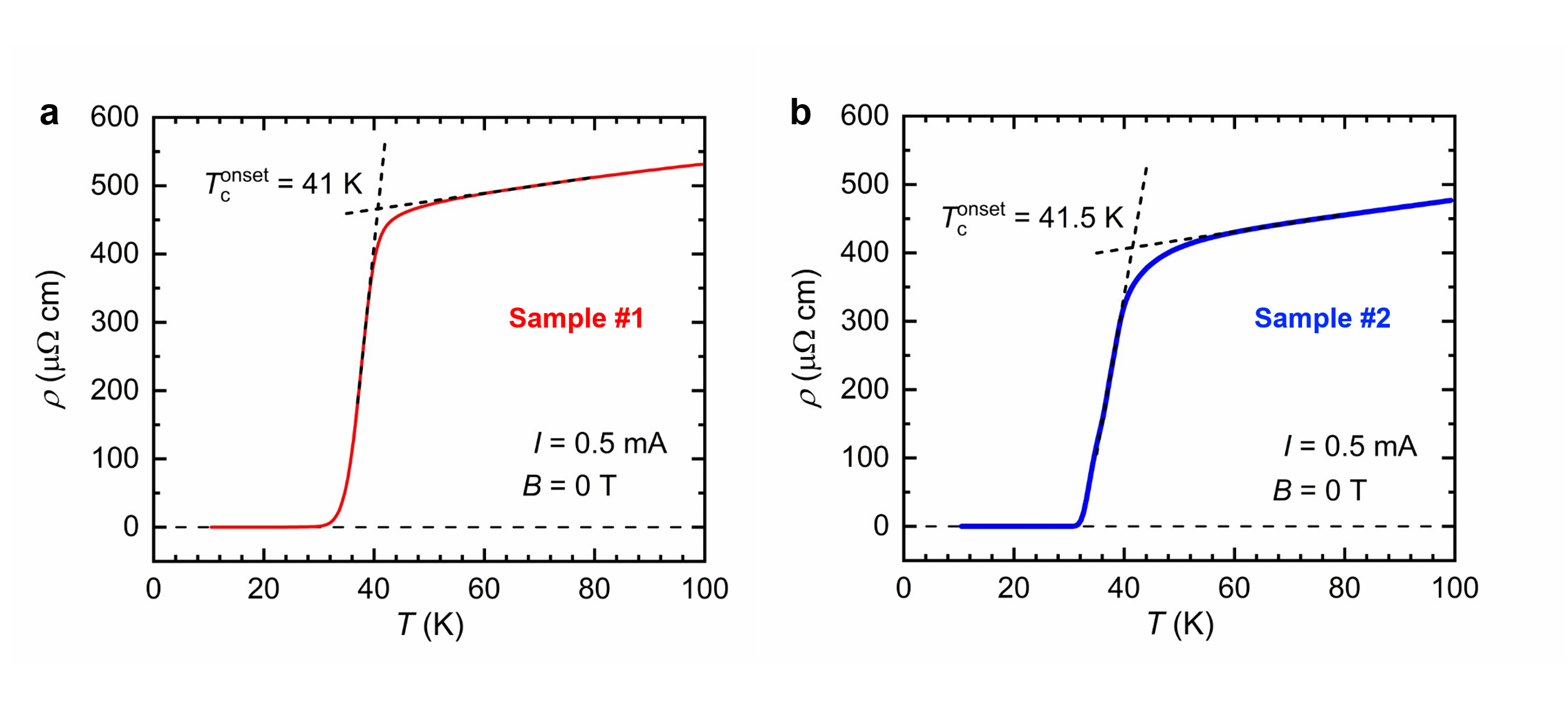


**Supplementary Figure S3 | Basic transport properties of the prepared samples.** Temperature dependence of the electrical resistivity *ρ* of (a) sample #1 (with a hydrogen concentration gradient) and (b) sample #2 (without a gradient) measured under dc current *I*_dc_ = 0.5 mA. The onset of the superconducting transition $T_{c}^{\mathrm{onset}}$ is evaluated from the intersection of two linear fits to the *ρ*–*T* curve (normal-state and transient-regime segments).


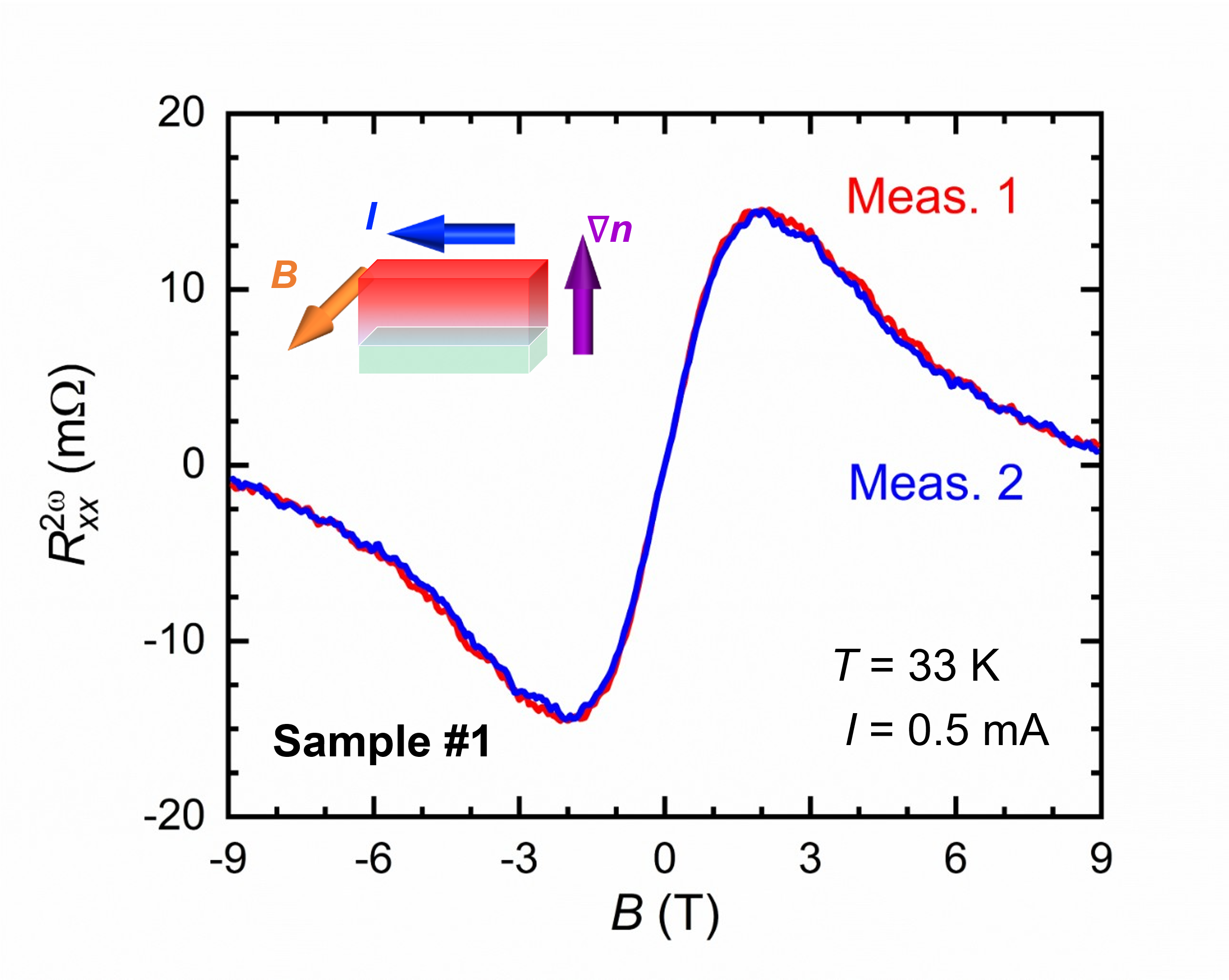


**Supplementary Figure S4 | Reproducibility of the nonreciprocal charge transport measurements.** Magnetic-field dependence of the second-harmonic magnetoresistance $R_{xx}^{2\omega}$ for sample #1, measured under *I*_ac_ = 0.5 mA at 33 K. The configuration of the magnetic field, electric current, and the direction of hydrogen concentration gradient is ***I*** ⊥ ***B*** ⊥ $\boldsymbol{\nabla}n_{H}$ as shown in the inset schematic. *Meas. 1* (red curve) corresponds to the data obtained in the initial measurement, whereas *Meas. 2* (blue curve) represents the data measured after removing the sample and remounting it.

**Supplementary References**

S1. G. L. J. A. Rikken, J. Fölling, P. Wyder, *Physical Review Letters* **2017**, *81*, 236602.

S2. G. L. J. A. Rikken, P. Wyder, *Physical Review Letters* **2005**, *94*, 016601.

S3. T. Ideue, K. Hamamoto, S. Koshikawa, M. Ezawa, S. Shimizu, Y. Kaneko, Y. Tokura, N. Nagaosa, Y. Iwasa, *Nature Physics* **2017**, *13*, 578.
